# Parietal Fast Sleep Spindle Density Decrease in Alzheimer's Disease and Amnesic Mild Cognitive Impairment

**DOI:** 10.1155/2016/8376108

**Published:** 2016-03-15

**Authors:** Maurizio Gorgoni, Giulia Lauri, Ilaria Truglia, Susanna Cordone, Simone Sarasso, Serena Scarpelli, Anastasia Mangiaruga, Aurora D'Atri, Daniela Tempesta, Michele Ferrara, Camillo Marra, Paolo Maria Rossini, Luigi De Gennaro

**Affiliations:** ^1^Department of Psychology, “Sapienza” University of Rome, 00185 Rome, Italy; ^2^Department of Physiology and Pharmacology, “Sapienza” University of Rome, 00185 Rome, Italy; ^3^Department of Biomedical and Clinical Sciences “Luigi Sacco”, University of Milan, 20157 Milan, Italy; ^4^Department of Life, Health and Environmental Sciences, University of L'Aquila, 67100 L'Aquila, Italy; ^5^Department of Biotechnological and Applied Clinical Sciences, University of L'Aquila, 67100 Coppito, Italy; ^6^Institute of Neurology, Catholic University of The Sacred Heart, 00168 Rome, Italy; ^7^IRCCS San Raffaele Pisana, 00163 Rome, Italy

## Abstract

Several studies have identified two types of sleep spindles: fast (13–15 Hz) centroparietal and slow (11–13 Hz) frontal spindles. Alterations in spindle activity have been observed in Alzheimer's disease (AD) and Mild Cognitive Impairment (MCI). Only few studies have separately assessed fast and slow spindles in these patients showing a reduction of fast spindle count, but the possible local specificity of this phenomenon and its relation to cognitive decline severity are not clear. Moreover, fast and slow spindle density have never been assessed in AD/MCI. We have assessed fast and slow spindles in 15 AD patients, 15 amnesic MCI patients, and 15 healthy elderly controls (HC). Participants underwent baseline polysomnographic recording (19 cortical derivations). Spindles during nonrapid eye movements sleep were automatically detected, and spindle densities of the three groups were compared in the derivations where fast and slow spindles exhibited their maximum expression (parietal and frontal, resp.). AD and MCI patients showed a significant parietal fast spindle density decrease, positively correlated with Minimental State Examination scores. Our results suggest that AD-related changes in spindle density are specific for frequency and location, are related to cognitive decline severity, and may have an early onset in the pathology development.

## 1. Introduction

Sleep spindles are transient waxing-waning events (11–15 Hz) that represent one of the principal electroencephalographic (EEG) hallmarks of nonrapid eye movements (NREM) sleep [1]. Spindle oscillations arise from the interaction between GABAergic inhibitory neurons of the thalamic reticular nucleus and thalamocortical networks [1–3]. Albeit their functional role is not clearly understood, many evidences suggest that sleep spindles may be involved in cortical development [4], sleep maintenance [3, 5], and cognitive functions including memory consolidation [6–10] and intellectual ability [10, 11].

Two main types of sleep spindles have been identified: fast (13–15 Hz) centroparietal spindles, with a source located in the precuneus, and slow (11–13 Hz) frontal spindles, with a source in the medial frontal region [12–14]. Albeit both kinds of spindles involve the activity of thalamus and superior temporal gyri, slow spindles are also related to the activity of the superior frontal gyrus, while fast spindles generation involves the activity of medial frontal cortex, hippocampus, and sensory-motor regions [14]. Fast parietal spindles seem to be involved in thalamocortical coupling [15], promoting the interaction between the hippocampus and the neocortex needed for memory consolidation [16–19]. The function of slow frontal spindles is not yet clear, albeit a role in corticocortical interactions has been proposed [15]. It should however be mentioned that others have questioned that a different generation mechanism is involved in the 11–13 Hz EEG activity [1]. According to this view, slower and faster sleep spindles frequencies have been attributed to a unitary mechanism, namely, the duration of the hyperpolarization-rebound sequence in the thalamocortical neurons: long hyperpolarization yielding slower, short hyperpolarization faster EEG frequencies (e.g., [20]). Cortical areas on which slow spindles have been detected are related to those thalamic nuclei in which the relay cells display long hyperpolarization (M. Steriade, personal communication; cit. in [1]).

Several evidences show that normal and pathological aging are characterized by changes in spindle activity [1, 21, 22]. In normal aging, a reduction of spindle density, amplitude, and duration has been observed [23–26]. Changes of spindle activity become progressively more evident as age increases [27, 28] and have been interpreted as an EEG index of the age-related modifications of sleep pattern [24, 27] and/or cognitive functioning [21]. In particular, reduced fast sleep spindles seem to be related to memory decline in older subjects [29]. As far as pathological brain aging is concerned, a decrease in spindle density and amplitude has been recently observed in patients with Parkinson's disease (PD) who developed dementia, compared with nondemented PD patients and healthy older subjects; moreover, the reduction of spindle amplitude in posterior regions was related to lower visuospatial abilities [30]. Notably, by separating fast and slow spindles, the authors found a selective decrease of fast spindles in demented PD patients, strongly linking fast spindles alterations and cognitive decline.

Alzheimer's disease, the most common age-related neurodegenerative disorder, is characterized by several sleep alterations [22]. Given the suggested role of sleep spindles in memory consolidation, intellectual abilities, and sleep maintenance, the study of spindle activity in AD patients should be of great interest. Nevertheless, only few have tried to examine in depth the AD-related sleep spindles alterations, showing in AD patients an exacerbation of the spindles changes found in the normal elderly population [22, 31–34]. Only Rauchs and coworkers [35] have assessed separately fast and slow spindles, reporting a selective decrease of fast sleep spindles in AD patients compared to normal elderly, and a positive correlation between fast spindle intensity (duration × amplitude) and impaired immediate recall in an episodic memory task. However, in this study, the topography of fast and slow spindles was not considered: both kinds of spindles were detected only at central derivations. Given the observations that fast and slow sleep spindles show different cortical topography and undergo a local mechanism of regulation [36, 37], the assessment of fast and slow spindles in the cortical regions where they exhibit their maximal expression is essential.

Since spindle changes characterize also normal aging, another important question concerns when, in the development of AD, a clear difference emerges between age-related and disease-related spindle alterations, and if these are linked with illness severity. This issue can be assessed by studying sleep spindles in people affected by amnesic Mild Cognitive Impairment (MCI), a preclinical stage of AD [38–40] characterized by memory impairment not reaching the criteria for the diagnosis of dementia [39]. Several sleep alterations have been observed in MCI patients [41, 42]. For what concern sleep spindles, Westerberg and coworkers [42] found a significant decrease of frontal (but not parietal) fast spindles in MCI patients compared with normal elderly, but this result is limited by a very small sample size (5 MCI patients). No studies have compared fast and slow spindles in AD versus MCI patients. In summary, the present literature suggests an alteration of fast rather than slow spindle oscillations in AD/MCI [35, 42], but the possible local specificity of this phenomenon, as well as its progression with illness severity, remains unclear. Moreover, while previous studies have assessed fast and slow spindle count [35, 42], to the best of our knowledge, fast and slow spindle density (number of spindles divided by NREM sleep duration) have never been considered in AD/MCI. This issue does not seem trivial due to the existence of polysomnographic differences (i.e., in the amount of NREM sleep) between AD/MCI and healthy controls.

With the aim to overcome some limits of the present literature on spindle activity in AD/MCI, we have assessed whole range, fast, and slow spindle density in AD, amnesic MCI, and healthy elderly controls (HC) in the cortical regions of their maximal expression, established by the empirical observation of their topography. Moreover, we have evaluated the possible relation between spindle density and cognitive impairment severity.

## 2. Materials and Methods

### 2.1. Subjects

In the present study, 15 AD patients (5 males and 10 females), 15 amnesic MCI patients (6 males and 9 females), and 15 healthy elderly control subjects (HC, 10 males and 5 females) were recruited. Demographic and clinical characteristics of the sample are reported in Table 1. Patients were selected among the elderly persons referred to the Neuropsychology Unit of the Gemelli Catholic University Hospital of Rome. HC were recruited in clubs for retired people. All subjects gave their written informed consent. The study was approved by the local Institutional Ethics Committee and was conducted in accordance with the Declaration of Helsinki.

### 2.2. Inclusion and Exclusion Criteria

All participants underwent cognitive screening by means of the Minimental State Examination (MMSE) [43]. Moreover, the State Trait Anxiety Index (STAI-Y1 and STAI-Y2) [44] and the Hamilton Depression Rating Scale (HDRS) [45] were administered in order to exclude major psychiatric illness.

Neuropsychological investigation for AD and MCI patients included a structured clinical evaluation, brain neuroimaging (MRI or CT), and a neuropsychological test battery for the assessment of specific cognitive functions such as memory, attention, executive function, visuoconstruction abilities, and language. In particular, memory assessment included Rey's Auditory Verbal Learning (RAVLT) [46], involving immediate recall (RAVLTir), delayed recall (RAVLTdr), and delayed recognition (RAVLTrec), delayed recall of the Rey figure [47], delayed recall of a three-word list [48], and delayed recall of a story [49, 50]. The functional status was assessed by the Activities of Daily Living/Instrumental Activities of Daily Living (ADL/IADL) questionnaire [51].

AD patients were included according to the National Institute on Aging-Alzheimer's Association workgroups [52] and DSM-IV criteria. People with amnesic MCI were enrolled according to guidelines and clinical standards [39, 53–56].

Common exclusion criteria for all participants were presence of neurological, psychiatric, or vascular disorders, obesity, and history of alcoholism or drug abuse. HC receiving psychoactive drugs were also excluded. The final enrollment in the study was based on the evaluation of regular sleep-waking cycle and on the absence of self-rated sleep disorders. The presence of other sleep disorders was objectively evaluated by nocturnal sleep recordings. In case of sleep disorder and/or respiratory diseases and obstructive sleep apnea syndrome (OSAS), subjects were excluded by subsequent analyses. Sleep quality and diurnal sleepiness of all participants were assessed by the Italian version of the Pittsburg Sleep Quality Index (PSQI, [57]), the Epworth Sleepiness Scale (ESS, [58]), and the Karolinska Sleepiness Scale (KSS, [59]).

### 2.3. Study Design

Participants underwent complete polysomnographic (PSG) recording of a single night of sleep. A Micromed system plus digital polygraph was used for the PSG recording. EEG signals were acquired with a sampling frequency of 256 Hz and bandpass filtered at 0.53–40 Hz. The 19 unipolar EEG derivations of the international 10–20 system (C3, C4, Cz, F1, F2, F3, F4, F7, F8, Fz, O1, O2, P3, P4, Pz, T3, T4, T5, and T6) were recorded from scalp electrodes with average mastoid references (A1 and A2), using Ag/AgCl electrodes. Electrooculogram (EOG) was recorded from electrodes placed about 1 cm from the medial and lateral canthi of the dominant eye. Electrocardiogram (EKG) and submental electromyogram (EMG) were also recorded. Finally, a pulse oximeter was placed on the right index finger with the aim to exclude sleep respiratory disorders. Impedance was kept below 5 KOhm.

### 2.4. Data Analysis

#### 2.4.1. Demographics and Clinical Characteristics

Age, years of education, and clinical characteristics (MMSE, HDRS, STAI Y-1, STAI Y-2, and PSQI scores) of AD, MCI, and HC were compared by means of one-way analyses of variance (ANOVAs), and* post hoc* comparisons were carried out by means of unpaired two-tailed *t*-tests. Alpha level was always set at 0.05.

#### 2.4.2. Sleep Measures

Sleep stages of the baseline (BSL) night were scored visually in 20 seconds epochs, according to standard criteria [60], excluding ocular and muscle artifacts. The following were considered as dependent variables: (a) stage 1 latency; (b) stage 2 latency; (c) total sleep time (TST), defined as the sum of time spent in stage 1, stage 2, SWS, and REM; (d) percentage of each sleep stage (time spent in a sleep stage/TST × 100); (e) wakefulness after sleep onset (WASO), in minutes; (f) number of awakenings; (g) number of arousals; (h) total bed time (TBT); and (i) sleep efficiency index (SEI = TST/TBT × 100). An awakening was scored whenever EEG/EMG activation occurred lasting more than 10 s. Arousals have been scored whenever EMG activation affected the EEG recording for periods shorter than 10 s.

The polysomnographic EEG measures were submitted to one-way ANOVAs comparing AD, MCI, and HC, and* post hoc *comparisons were carried out by means of unpaired two-tailed *t*-tests.

#### 2.4.3. Spindle Detection and Analysis

Spindle detection was performed by means of a customized algorithm in MATLAB [61–64]. NREM epochs were bandpass-filtered between 11 and 15 Hz (–3 dB at 10 and 16 Hz) using a Chebyshev Type II filter. The detection of a spindle occurred when the mean signal amplitude of each channel exceeded an upper threshold set at 6 times the mean single channel amplitude. The local amplitude maximum above the upper threshold was considered as the peak amplitude of the single spindle. The points at which the amplitude fell below a lower threshold (2 times the mean amplitude of each channel) occurring at least 0.25 s from the peak were considered as the beginning and the end of the spindle (maximum duration: 1.5 s). Spindles falling within the 11–13 Hz frequency range were considered as “slow,” while those falling in the 13–15 Hz range were considered as “fast.” Spindle density was calculated as the number of spindles divided by artifact-free NREM sleep minutes. The EEG channels in which the maximum mean sleep density was detected for fast and slow spindles were considered for the statistical analysis. Specifically, the maximum mean fast spindle density was observed at Pz, while the slow spindles showed two similar maximum mean density values at F3 and F4. For these derivations, group differences in spindle density (for the whole spindle range and separately for fast and slow spindles) were assessed by means of one-way ANOVAs comparing AD, MCI, and HC, and* post hoc *comparisons were carried out by means of unpaired two-tailed *t*-tests. Preliminary analyses have also considered sex as a between factor, without any significant main effect or interaction involving this factor. For this reason, it was collapsed in the subsequent analyses.

Finally, in case of significant difference in whole range, fast, or slow spindle density between groups on a specific cortical derivation, Pearson's correlation coefficient was computed between spindle density in that derivation and MMSE scores, in order to assess the relationship between sleep spindles and cognitive impairment.

## 3. Results

### 3.1. Demographic and Clinical Characteristics

Results of the one-way ANOVAs and relative* post hoc t*-tests performed on demographic and clinical characteristics of AD, MCI, and HC are reported in Table 1. No significant age or education difference has been observed. MMSE scores were significantly different between the three groups:* post hoc t*-tests show significantly higher MMSE scores in HC compared with AD and MCI; moreover, MMSE scores were significantly higher in MCI compared with AD. A significant difference has been observed also for STAI Y-1 scores:* post hoc t*-tests show higher state anxiety in AD patients, compared with MCI patients and HC. No significant difference has been observed for STAI Y-2, HDRS, and PSQI.

### 3.2. Sleep Measures

Results of the one-way ANOVAs and* post hoc t*-tests performed on PSG measures are reported in Table 2. A significant difference has been found for SWS, and* post hoc t*-tests show a higher percentage of SWS in HC compared to AD.

### 3.3. Topographical Distribution of Spindle Density

**Figure 1:**
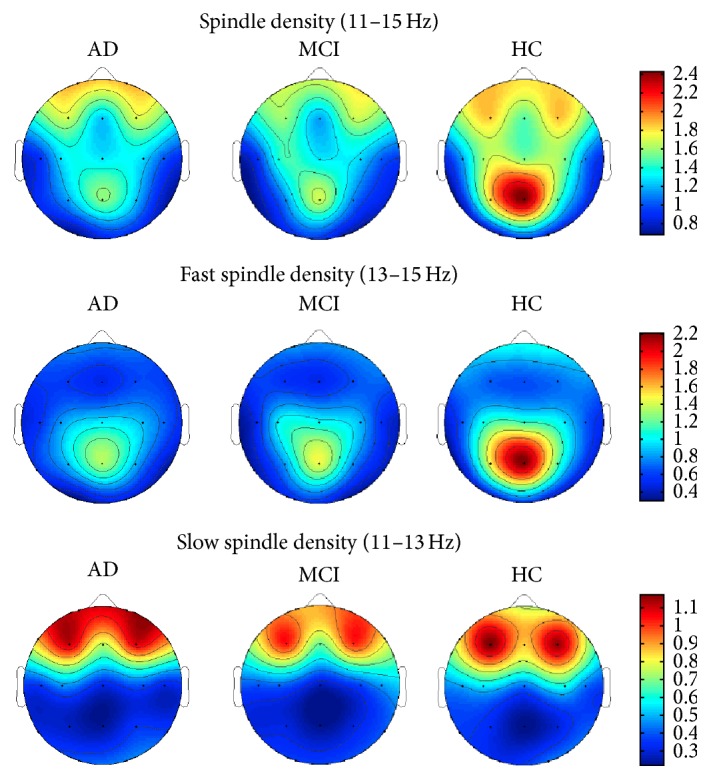
Whole range (11–15 Hz), fast (13–15 Hz), and slow (11–13 Hz) spindle density topographical scalp maps in AD patients, amnesic MCI patients, and HC. The maps are based on the 19 derivations of the 10–20 system (electrodes positions indicated by black dots). Values are color-coded and plotted at the corresponding position on the planar projection of the hemispheric scalp model. Values between electrodes were interpolated (biharmonic spline interpolation). Values are expressed in terms of number of spindles divided by artifact-free NREM sleep minutes.

Figure 1 depicts the whole range (11–15 Hz), fast (13–15 Hz), and slow (11–13 Hz) spindle density topographical scalp maps in AD, MCI, and HC.

Considering the whole spindle range, a parietal predominance of spindle density (particularly in correspondence of the midline derivation) can be observed in HC, followed by two density peaks in the left and right frontal areas. Also, AD and MCI patients show a midline parietal and two frontal peaks, but with a generalized reduction of spindle density, in particular in the parietal region.

Fast spindle density shows a clear maximum peak in the parietal region in correspondence with the midline in all of the 3 groups, albeit this peak progressively decreases in MCI and AD patients.

The 3 groups show a similar topographical distribution of slow sleep spindles, with two density peaks in correspondence with the left and right frontal areas.

### 3.4. Changes in Spindle Density in AD and MCI

**Figure 2:**
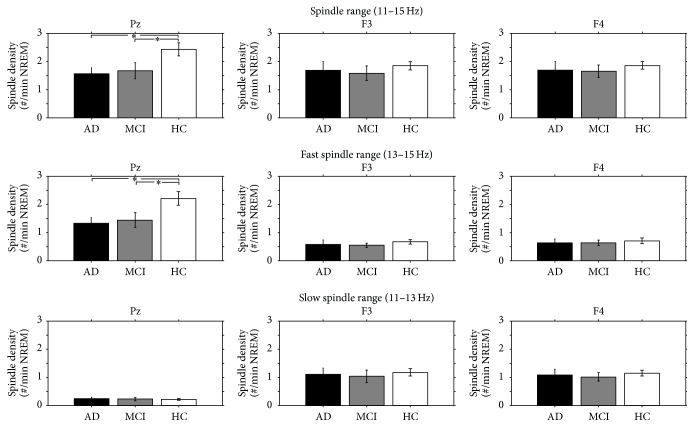
Whole range (11–15 Hz), fast (13–15 Hz), and slow (11–13 Hz) spindle density of AD patients (black bars), amnesic MCI patients (gray bars), and HC (white bars) in the cortical derivations where the maximum mean density values were observed for fast (Pz) and slow (F3 and F4) spindles. Error bars represent the standard errors. Asterisks indicate between-groups statistically significant differences (*p* ≤ 0.05) after* post hoc *unpaired *t*-tests.

Figure 2 illustrates the comparisons of spindle density (whole range, fast, and slow) of AD, MCI, and HC in the cortical derivations where the maximum mean density values were observed (Pz for fast spindles; F3 and F4 for slow spindles).

The results of the one-way ANOVAs show a significant difference in the whole range spindle density (*F*_2,42_ = 3.67, *p* = 0.03) and fast spindle density (*F*_2,42_ = 4.11, *p* = 0.02) at Pz, without difference in slow spindle density (*F*_2,42_ = 0.04, *p* = 0.96).* Post hoc t*-tests show a significantly higher whole range spindle density in HC compared to AD (*t* = 2.74, *p* = 0.01) and MCI (*t* = 2.07, *p* = 0.05), without differences between AD and MCI (*t* = 0.30, *p* = 0.77), and a significantly higher fast spindle density in HC compared to AD (*t* = 2.80, *p* = 0.009) and MCI (*t* = 2.15, *p* = 0.04) without differences between AD and MCI (*t* = 0.36, *p* = 0.72). No significant differences have been found on F3 (whole range spindle density: *F*_2,42_ = 0.28, *p* = 0.76; fast spindle density: *F*_2,42_ = 0.33, *p* = 0.72; slow spindle density: *F*_2,42_ = 0.13, *p* = 0.88) and F4 (whole range spindle density: *F*_2,42_ = 0.22, *p* = 0.80; fast spindle density: *F*_2,42_ = 0.15, *p* = 0.86; slow spindle density: *F*_2,42_ = 0.17, *p* = 0.84).

### 3.5. Correlation between Spindle Density and Cognitive Impairment

**Figure 3:**
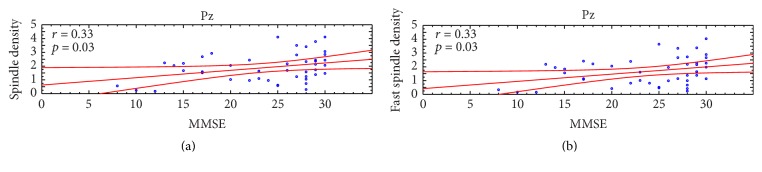
Scatterplots of the individual correlations between Minimental State Evaluation (MMSE) scores and (a) whole range (11–15 Hz) and (b) fast (13–15 Hz) spindle density at Pz cortical derivation (*p* ≤ 0.05). Pearson's *r* and relative *p* values are reported for each scatterplot.

Since significant between-group differences in whole range and fast spindle density have been observed at Pz, correlations between spindle density and degree of cognitive impairment have been assessed only in this derivation.  Figure 3 depicts the scatterplots of the correlations between MMSE scores and whole range spindle (a) and fast spindle density (b) on Pz, showing that whole range and fast spindle density were positively correlated (whole range spindle density: *r* = 0.33, *p* = 0.03; fast spindle density: *r* = 0.33, *p* = 0.03) with MMSE scores.

## 4. Discussion

The present study assessed for the first time the presence of topographic differences in spindle activity in AD, MCI, and healthy elderly controls. Results revealed in AD and MCI patients a reduction of the whole range spindle density over the midline parietal area compared to HC. This difference can be ascribed to a significant decrease of fast parietal spindle density, while no significant alteration in slow spindle density has been observed, not even in the cortical area where slow spindles exhibit their maximum density peak (i.e., the frontal region). Furthermore, both whole range and fast parietal spindle density were positively correlated with MMSE scores.

Our findings confirm that the classically described topographical distribution of fast and slow sleep spindles (with parietal and frontal maxima, resp.) can be observed also in AD, MCI, and healthy older subjects. A reduction of sleep spindles in AD patients has been previously found [31–34], but only two studies have assessed separately sleep spindles with different frequency ranges, finding a selective decrease of the number of fast spindles in AD [35] and MCI [42]. Our results strengthen these evidences, showing that (1) not only fast spindles count but also fast spindle density is reduced in AD/MCI; (2) this reduction occurs specifically in the cortical region in which fast spindles show their maximum density, that is, the midline parietal area, empirically assessed; (3) parietal fast spindle density is positively correlated with cognitive status; and (4) spindle density does not differ between AD and MCI. In line with our results, Latreille and coworkers [30] have recently found a generalized reduction of fast spindle density in patients with PD who developed dementia, compared with nondemented PD patients and normal older subjects. Altogether, these findings suggest the existence of a strong and selective relation between fast spindles alteration and cognitive decline in demented patients. Moreover, the present results suggest that pathology-related spindles alterations may arise in an early stage of disease, since MCI and AD patients show a similar spindle density decrease, compared with HC.

Several studies suggested a role of sleep spindles in memory consolidation [10, 21], intellectual abilities [10], sleep maintenance [3, 5], and synaptic plasticity [65, 66]. In particular, fast spindles seem to be involved in sleep-dependent procedural [67–70] and declarative learning [71]. A fast prefrontal spindle density decrease related to impaired episodic learning and hippocampal activity has been recently observed in older adults, compared with young subjects [29]. In addition, Rauchs and coworkers [35] reported a relation between fast central spindle intensity and impaired immediate episodic recall in AD patients. In demented PD patients (compared with nondemented PD patients and normal elderly), a relation between decreased spindle amplitude in posterior regions and lower visuospatial abilities has been observed [30]. These studies refer to different conditions (normal aging, AD, and demented PD) and found several specific spindle alterations in relation to specific cognitive dysfunctions, with different topographical specificity. However, taken together, these findings suggest a relation between altered spindle (mainly fast) activity and impaired cognitive functioning (and in particular memory processes) in normal and pathological aging. In this view, alterations in spindle activity may be considered as a marker of cognitive deterioration, not representative of a specific neurodegenerative disease. The topography of such alterations may be relevant for the discrimination of different conditions characterized by neurodegenerative processes. To assess this hypothesis, future studies should compare the topography of spindle activity in different neurodegenerative diseases. For what concern AD/MCI patients, our findings confirm the existence of a selective relation between fast spindle alterations and general cognitive impairment, and this relation is specific for the parietal (rather than frontal) cortical region.

Sleep spindles are generated by the GABAergic cells in the thalamic reticular nucleus [65, 72, 73]: their repetitive spike-bursts induce rhythmic inhibitory postsynaptic potentials in the thalamocortical network; the postinhibitory spike-burst activity of the glutamatergic neurons produces excitatory postsynaptic potentials at a cortical level. This mechanism seems to support long-term potentiation and, as a consequence, memory consolidation [21]. Thalamic alterations have been observed in AD [74–80] and MCI [79, 80]. It is possible that thalamic damage may account for the spindle density decrease and its relation with impaired cognitive functioning in AD/MCI patients. Moreover, Rauchs and coworkers [35] suggest that fast spindle alterations in AD patients may be explained by the relation existing between fast sleep spindles and hippocampal activity. In fact, fast spindles generation involves hippocampal activation and hippocampal-cortical connectivity [14, 81]. Moreover, a close temporal association between sleep spindles and hippocampal ripples (high-frequency oscillations) has been observed in rodents [18, 82] and a recent human study have found that parahippocampal ripples were primarily coupled with parahippocampal and fast parietal spindles, and only secondarily with slow frontal spindles [83]. This spindle-ripple association is considered as an important mechanism for the communication between hippocampus and neocortex, finalized to sleep-dependent memory consolidation [16–19]. Coherently, the age-related fast sleep spindles decrease predicts impaired next day episodic learning and hippocampal activation [29]. The strong and early hippocampal damage in AD patients is well known [74, 76, 84], and hippocampal alterations have been observed also in MCI patients [80, 85]. The selective decrease of fast sleep spindles and its relation with cognitive decline in AD/MCI, then, may also be explained in relation to the hippocampal damage. Future neuroimaging studies should directly assess this hypothesis relating EEG and MRI measures in AD/MCI.

## 5. Conclusion

Our results shed light on sleep spindle changes in AD and amnesic MCI patients, showing a selective parietal fast spindle density decrease in these patients compared with HC, in relation to decreased cognitive functioning. These findings suggest that AD-related spindle density changes are specific for frequency (regarding fast rather than slow spindles) and location (parietal rather than frontal), may have an early onset (MCI and AD patients show a similar spindle density decrease), and are related to the severity of cognitive impairment. Recently, alteration in spindle activity has been suggested as a possible biomarker for (1) regional brain aging [25], (2) future neurodegeneration in REM sleep behavior disorder [86], and (3) cognitive decline in demented PD patients [30]. Our results are in line with the view of altered sleep spindle activity as a marker of cognitive deterioration. However, more types of evidence are needed to better understand the relation between regional changes in spindle characteristics, specific cognitive impairment, and neurological damage in AD/MCI, in order to further characterize the difference between age-related and pathology-related alteration in sleep spindles.

## Figures and Tables

**Table 1:** Mean and standard errors (SE) of demographic (age, education) and clinical (Minimental State Examination (MMSE) scores, Hamilton Depression Rating Scales (HDRS) scores, State Trait Anxiety Index (STAI Y-1 and STAI Y-2) scores, Pittsburg Sleep Quality Index (PSQI) scores) characteristics of AD patients, amnesic MCI patients, and HC. The results of the one-way ANOVAs (*F* and *p* values) were also reported, with *post hoc *unpaired *t*-test (*p* values) when ANOVAs were significant (*p* ≤ 0.05). Significant between-groups differences are indicated in bold.

Variables	AD	MCI	HC	*F*_2,42_	*p*	AD versus MCI	AD versus HC	MCI versus HC
	Mean (SE)	Mean (SE)	Mean (SE)					
Age (years)	70.80 (2.40)	71.10 (2.28)	70.80 (1.63)	0.005	0.99	—	—	—
Education (years)	9.4 (1.49)	12.4 (1.13)	11.8 (1.24)	1.51	0.23	—	—	—
MMSE	16.07 (1.10)	26.07 (0.53)	29.07 (0.27)	**89.32**	**<0.0001**	**<0.0001**	**<0.0001**	**<0.0001**
HDRS	9.62 (1.52)	7.87 (1.03)	6.33 (0.83)	2.07	0.14	—	—	—
STAI Y-1	40 (2.70)	32.87 (1.66)	32.33 (1.63)	**4.37**	**0.02**	**0.03**	**0.02**	0.82
STAI Y-2	41.92 (2.54)	38.4 (2.94)	33.8 (2.25)	2.40	0.10	—	—	—
PSQI	4.79 (0.65)	5.13 (0.99)	5.27 (0.50)	0.11	0.9	—	—	—

**Table 2:** Mean and standard errors of the polysomnographic variables of AD patients, amnesic MCI patients, and HC. The results of the one-way ANOVAs (*F* and *p* values) were also reported, with *post hoc *unpaired *t*-test (*p* values) when ANOVAs were significant (*p* ≤ 0.05). Significant between-groups differences are indicated in bold.

Variables	AD	MCI	HC	*F*_2,42_	*p*	AD versus MCI	AD versus HC	MCI versus HC
	Mean (SE)	Mean (SE)	Mean (SE)					
Stage 1 latency (min)	41.04 (9.78)	27.44 (4.88)	19.58 (5.86)	2.30	0.11	—	—	—
Stage 2 latency (min)	33.27 (8.61)	26.61 (4.32)	13.29 (4.58)	2.73	0.08	—	**—**	**—**
Stage 1 (%)	13.33 (3.06)	9.34 (1.44)	6.55 (1.33)	2.63	0.08	—	**—**	—
Stage 2 (%)	75.92 (3.33)	76.21 (1.88)	77.91 (2.10)	0.18	0.83	—	—	—
SWS (%)	0.08 (0.05)	0.14 (0.08)	0.79 (0.34)	**3.73**	**0.03**	0.53	**0.05**	0.07
REM (%)	10.66 (2.78)	14.28 (1.85)	15.22 (1.53)	1.29	0.28	—	—	—
WASO (min)	92.24 (14.25)	100.89 (14.06)	90.37 (8.98)	0.20	0.82	—	—	—
Awakenings (#)	18.13 (3.87)	21.33 (2.13)	20.13 (2.23)	0.32	0.73	—	—	—
Arousals (#)	40.00 (9.53)	32.73 (6.53)	34.40 (7.96)	0.22	0.80	—	—	—
TST (min)	263.82 (22.46)	274.31 (16.02)	303.36 (17.22)	1.19	0.31	—	—	—
TBT (min)	388.09 (20.91)	401.47 (9.60)	406.82 (13.52)	0.39	0.68	—	—	—
SEI% (TST/TBT)	67.56 (4.15)	68.31 (3.61)	74.13 (2.98)	0.99	0.38	—	—	—

SWS, slow-wave sleep; REM, rapid eye movement; WASO, waking after sleep onset; TST, total sleep time; TBT, total bed time; SEI, sleep efficiency index.
